# Puncture of the Colon in Tympanitis (Selected.)

**Published:** 1872-01

**Authors:** 


Puncture of the Colon in Tympanitis.—Mr. J. H. Wathen refers, in the British Medical Journal for October, 1871, to six cases in which puncture of the colon was resorted to for the relief of tympanitis. In one case, the distension of the bowels came on during peritonitis, and, although temporary relief was procured by the operation, the patient ultimately sank. In the second case death was due to double pneumonia. In the third and fourth cases the cause of death is not stated, but the patients lived several days after the operation. In the fifth case the patient recovered; and in the sixth case the patient recovered sufficiently to go upon a drunken spree, of the effects of which he died.

